# Knowledge-Practice Performance Gap in Clinical Large Language Models: Systematic Review of 39 Benchmarks

**DOI:** 10.2196/84120

**Published:** 2025-12-01

**Authors:** Eun Jeong Gong, Chang Seok Bang, Jae Jun Lee, Gwang Ho Baik

**Affiliations:** 1 Department of Internal Medicine Hallym University College of Medicine Chuncheon, Gangwon Republic of Korea; 2 Institute for Liver and Digestive Diseases Hallym University Chuncheon, Gangwon-do Republic of Korea; 3 Institute of New Frontier Research Hallym University College of Medicine Chuncheon, Gangwon Republic of Korea; 4 Department of Anesthesiology and Pain Medicine Hallym University College of Medicine Chuncheon, Gangwon Republic of Korea

**Keywords:** large language model, benchmark, artificial intelligence, clinical medicine, LLM

## Abstract

**Background:**

The evaluation of large language models (LLMs) in medicine has undergone a shift from knowledge-based testing to practice-based assessment, representing an evolution in how we measure artificial intelligence readiness for clinical deployment. While LLMs now routinely exceed human performance on medical licensing examinations, their translation to clinical practice remains poorly characterized.

**Objective:**

This systematic review aims to categorize and analyze medical LLM benchmarks, examining performance patterns across different evaluation paradigms and identifying gaps in current assessment methodologies.

**Methods:**

The protocol was registered at PROSPERO (CRD420251139729). Four databases (MEDLINE/PubMed, Embase/Ovid, Cochrane Library, and arXiv) were searched from inception to August 31, 2025, using keywords related to clinical medicine benchmarks in LLMs. Studies were included if they (1) investigated clinical medicine benchmarks in LLMs, (2) were published in English, and (3) were available in full-text. Studies were excluded if they evaluated nonmedical domains or lacked benchmark validation. Methodological quality was assessed using the Mixed Methods Appraisal Tool (version 2018) by 2 independent reviewers (κ=0.91). Due to heterogeneity in evaluation metrics preventing meta-analysis, narrative synthesis was conducted using structured categorization of benchmark types.

**Results:**

From 3917 screened records, 39 medical LLM benchmarks were identified and categorized into 21 (54%) knowledge-based, 15 (38%) practice-based, and 3 (8%) hybrid frameworks. These benchmarks collectively encompass over 2.3 million questions across 45 languages and 172 medical specialties. Traditional knowledge-based benchmarks show saturation with leading models achieving 84%-90% accuracy on USMLE (United States Medical Licensing Examination)–style examinations, approaching or exceeding average physician performance. However, practice-based assessments reveal performance challenges, with specific benchmarks showing varied results: DiagnosisArena 45.82% (95% CI 42.9%-48.8%), MedAgentBench 69.67% (95% CI 64.2%-74.6%), and HealthBench 60% (95% CI 58.6%-61.3%) success rates, with practice-based benchmarks showing lower performance (45%-69%) compared to knowledge benchmarks (84%-90%). Task-specific analysis revealed differential performance patterns: factual retrieval maintained 85%-93% accuracy, clinical reasoning dropped to 50%-60%, diagnostic tasks achieved 45%-55% success, and safety assessment showed significant gaps at 40%-50% accuracy despite being life-critical. Geographic representation spans 6 continents with 18 (46%) benchmarks, incorporating non-English content. Quality assessment revealed 26% (10/39) of benchmarks had insufficient methodological reporting for complete evaluation.

**Conclusions:**

This systematic review provides the first comprehensive analysis quantifying the significant “knowledge-practice gap” in medical artificial intelligence: high performance on knowledge-based examinations (84%-90%) does not translate to clinical competence (45%-69%), with safety assessments at 40%-50%. Our findings provide quantitative evidence for regulators and health systems that examination scores are insufficient and misleading proxies for clinical readiness. This review concludes that autonomous deployment is not currently justifiable and that all evidence-based implementation strategies must mandate practice-oriented validation and robust human-in-the-loop oversight to ensure patient safety.

**Trial Registration:**

PROSPERO CRD420251139729; https://www.crd.york.ac.uk/PROSPERO/view/CRD420251139729

## Introduction

The integration of artificial intelligence (AI) into clinical medicine has reached a transition point. Large language models (LLMs) now routinely exceed human performance on medical licensing examinations, with OpenAI’s o1-preview model achieving 96% accuracy on the MedQA dataset—a benchmark derived from USMLE (United States Medical Licensing Examination) questions [1-3]. Yet, this achievement raises an important question: does passing medical examinations equate to competent clinical practice? Despite these impressive examination scores, real-world clinical deployment remains limited. A systematic review of LLM applications in clinical workflows identified only 4 peer-reviewed studies documenting actual implementation, all published between 2024-2025 [4]. Moreover, analysis of 1016 Food and Drug Administration (FDA)–authorized AI or machine learning medical devices revealed that no devices using LLMs have received regulatory clearance as of 2024 [5]. This regulatory void persists despite rapid commercial deployment, highlighting an important gap between market availability and evidence-based validation.

While multiple approaches exist for evaluating LLM performance—including statistical metrics (BLEU [bilingual evaluation understudy], ROUGE [Recall-Oriented Understudy for Gisting Evaluation], perplexity, mean reciprocal rank, BERTScore [Bidirectional Encoder Representations From Transformers Score], etc) [6], human evaluation, model-based evaluation (LLM-as-a-judge), and various evaluation frameworks—benchmarks have emerged as the dominant methodology in medical AI assessment [7]. This preference stems from several advantages: benchmarks provide standardized, reproducible comparisons across models and time points; they enable objective quantification of progress against established baselines; they offer cost-effective evaluation at scale without requiring extensive human resources; and most importantly, they facilitate regulatory approval processes that demand consistent, validated metrics [8,9]. Unlike subjective human evaluation or computationally expensive model-based approaches, benchmarks deliver immediate, interpretable results that can guide both development priorities and clinical deployment decisions. The medical community’s familiarity with standardized testing through licensing examinations further reinforces benchmark adoption, making performance metrics such as “passing USMLE” immediately meaningful to clinicians and administrators alike [2,3,10].

This question has driven a shift in medical AI evaluation. Traditional benchmarks, which dominated the field from 2020 to 2024, focused primarily on testing medical knowledge through multiple-choice questions [11,12]. These assessments, while valuable for establishing baseline capabilities, fail to capture the complexity of real clinical encounters. Of interest, evaluating artificial systems on traditionally human qualities such as empathy—asking “Doctor, what is your empathy or patient satisfaction score?”—may interestingly better reflect comprehensive health care outcomes than pure knowledge assessment, as these metrics correlate with tangible patient benefits, including improved diabetic control and reduced postoperative pain [13]. Recent evidence quantifies this knowledge-practice performance gap. Studies using conversational frameworks demonstrate diagnostic accuracy drops from 82% on traditional case vignettes to 62.7% on multiturn patient dialogues, a 19.3 percentage point decrease [14]. A systematic review of 761 LLM evaluation studies revealed only 5% assessed performance on real patient care data, with the vast majority relying on medical examination questions that may not reflect actual clinical competence [7]. This disconnect is further evidenced by randomized trials showing GPT-4 availability to physicians did not significantly improve clinical reasoning despite the AI demonstrating superior standalone diagnostic performance [15].

The 2025 introduction of practice-based evaluation frameworks marks a significant development in medical AI assessment. HealthBench by OpenAI evaluates LLMs through 5000 multiturn conversations simulating real clinical interactions, with evaluation criteria developed by 262 physicians across 26 specialties [16]. Similarly, the Clinical Safety-Effectiveness Dual-Track Benchmark (CSEDB) introduces 2069 clinical vignettes tested against 17 safety criteria and 13 effectiveness criteria, revealing that while general models excel at routine cases, they demonstrate important gaps in specialized scenarios such as drug interactions [17]. This shift from testing what AI knows to how AI practices medicine reflects a growing recognition that clinical competence extends far beyond factual knowledge to encompass reasoning, communication, uncertainty management, and contextual adaptation [18]. Patient safety concerns compound these performance limitations. Large-scale evaluation of LLM-generated clinical notes identified hallucination rates with significant proportions classified as errors that could impact patient management [19]. Analysis of FDA adverse event reports documented hundreds of adverse events involving AI- or machine learning–enabled medical devices [20]. These findings underscore the necessity of rigorous validation and human oversight in clinical deployment.

Paradoxically, while LLMs demonstrate important gaps in clinical reasoning and safety, they consistently outperform physicians on empathy metrics in blinded evaluations [13,21]. This “empathy paradox” raises questions about evaluation priorities, as empathy correlates with improved patient outcomes, including better glycemic control and reduced complications [22], yet may mask underlying clinical competence deficits.

For clinicians, understanding these evaluation frameworks has become essential. As AI systems increasingly participate in clinical decision-making, practitioners must be able to critically evaluate benchmark performance, understand the limitations of different assessment methodologies, and make informed decisions about when and how to integrate these tools into patient care [23,24]. This systematic review examines medical LLM benchmarks, categorizes evaluation approaches into knowledge-based and practice-based approaches, analyzes their performance patterns, and identifies major gaps in current evaluation methodologies to inform clinical AI implementation. Therefore, the objectives of this systematic review are to (1) identify all benchmarks used to evaluate LLM performance in clinical medicine, (2) characterize their methodological approaches, domains, and validation, (3) assess their quality using standardized criteria, and (4) identify gaps and priorities for future benchmark development.

## Methods

### Study Design

A systematic review was conducted following the PRISMA (Preferred Reporting Items for Systematic Reviews and Meta-Analyses) and PRISMA-S (an extension to the Preferred Reporting Items for Systematic Reviews and Meta-Analyses Statement for Reporting Literature Searches in Systematic Reviews) guidelines [25,26] (Multimedia Appendices 1 and 2), and the protocol of this systematic review was prospectively developed and subsequently registered at PROSPERO (CRD420251139729; registration date: September 3, 2025) following protocol finalization. The full protocol is available on the web. Three amendments were made to the registered protocol during review conduct: (1) arXiv was added as a supplementary database to capture rapidly published preprints in this fast-evolving field, (2) the Mixed Methods Appraisal Tool (MMAT) replaced the originally planned QUADAS-2 (Quality Assessment of Diagnostic Accuracy Studies, Version 2) to better accommodate the heterogeneous study designs of benchmark development papers, and (3) the search end date was extended from June 2025 to August 2025 to maximize currency of included benchmarks.

### Searching Strategies

We searched MEDLINE via PubMed [27], Embase via Ovid [28], Cochrane Library via Wiley [29], and arXiv [30] from inception to August 31, 2025. The complete search strategies for all databases are provided in Textbox 1. Language was restricted to English in MEDLINE and Embase, with additional publication type limits in Embase (articles, articles in press, and reviews). We performed backward citation searching by screening reference lists of all included studies and relevant systematic reviews. Forward citation searching was not conducted. We did not contact experts or manufacturers. The search was not peer reviewed. Duplicates were removed manually using EndNote X20 (Clarivate Analytics 2020).

The search strategy combined MeSH (Medical Subject Headings) terms and free-text keywords including: (“large language model” OR “LLM” OR “GPT” OR “ChatGPT” OR “artificial intelligence” OR “machine learning”) AND (“benchmark” OR “evaluation” OR “assessment” OR “performance metric”) AND (“medical” OR “clinical” OR “healthcare” OR “medicine”). A broad search strategy was used to maximize sensitivity in capturing all relevant medical LLM benchmarks, accepting a higher screening burden to ensure comprehensive coverage of this rapidly evolving field.

**Searching strategy to find the relevant papers.**

**Database: MEDLINE (through PubMed)**

- #1 “large language model[tiab]” OR “LLM[tiab]” OR “GPT[tiab]” OR “ChatGPT[tiab]” OR “artificial intelligence[tiab]” OR “machine learning[tiab]”: 233558
- #2 “benchmark[tiab]”: 215
- #3 #1 AND #2: 4
- #4 #3 AND English[Lang]: 4

**Database: Embase (through OVID)**

- #1 'large language model':ab,ti,kw OR 'LLM':ab,ti,kw OR 'GPT':ab,ti,kw OR 'ChatGPT':ab,ti,kw OR 'artificial intelligence':ab,ti,kw OR 'machine learning':ab,ti,kw: 272365
- #2 'benchmark':ab,ti,kw: 60660
- #3 #1 AND #2: 4643
- #4 #3 AND ([article]/lim OR [article in press]/lim OR [review]/lim) AND [English]/lim: 3834

**Database: Cochrane Library**

- #1 'large language model':ab,ti,kw OR 'LLM':ab,ti,kw OR 'GPT':ab,ti,kw OR 'ChatGPT':ab,ti,kw OR 'artificial intelligence':ab,ti,kw OR 'machine learning':ab,ti,kw: 17124
- #2 'benchmark':ab,ti,kw 1226
- #3 #1 AND #2: 75

**Database: arXiv**

- #1 'large language model'(all field):57759
- #2 'benchmark'(all field): 104172
- #3 'clinical' OR 'medical'(all field): 4120
- #4 #1 AND #2 #3: 597

### Study Selection Criteria

Studies were included if they satisfied the following criteria: (1) any type of study that investigated medical benchmarks in the LLMs field, (2) studies published in English, and (3) studies available in full-text format. The exclusion criteria were as follows: (1) studies that did not report medical benchmarks in the LLMs field, and (2) studies that reported benchmarks from nonmedical field areas. Two independent reviewers (EJG and CSB) screened titles and abstracts, followed by full-text review, with disagreements resolved through consensus or third-party arbitration.

### Data Extraction and Analysis

Data were extracted independently by 2 reviewers (EJG and CSB) using a standardized form piloted on 5 studies (κ=0.91, 95% CI 0.65-0.99). For multiple reports of the same benchmark, we used the most comprehensive publication as the primary source. We systematically reviewed traditional medical benchmarks (ie, MedQA, MedMCQA, PubMedQA, etc), analyzed the emergence of practice-based evaluation frameworks such as HealthBench and CSEDB, and examined direct comparisons between LLMs and physicians across multiple performance dimensions, including technical accuracy and empathy metrics. Data extraction included benchmark characteristics (dataset size, question types, and evaluation metrics), model performance scores, validation methodologies, and clinical relevance assessments.

### Methodological Quality Assessment

Quality assessment of individual studies was performed using the MMAT for diverse study designs [31], given the heterogeneity of included studies ranging from technical validation papers to clinical comparison trials. The MMAT evaluates methodological quality through specific criteria: S1 and S2 assess whether research questions are clear and whether the data address these questions. For quantitative descriptive studies (criteria 4.1-4.5), the tool evaluates: of 4.1, sampling strategy relevance—whether the sampling approach aligns with study objectives; for benchmark studies, this includes whether question selection methods (random sampling, expert curation, and stratified by specialty) are justified and documented; of 4.2, sample representativeness—whether the sample reflects the target population; we assessed whether benchmark questions adequately represent the breadth of clinical practice, specialty distributions, and difficulty levels they claim to evaluate; of 4.3, measurement appropriateness—whether data collection methods are suitable for the research questions; for benchmarks, we evaluated whether evaluation metrics (accuracy, *F*_1_-score, and area under the curve), ground truth labeling procedures, and validation methods are appropriate for assessing LLM clinical competence; of 4.4, nonresponse bias—whether response rates are acceptable and nonrespondents are adequately described; in the benchmark context, this criterion assessed whether studies reported model evaluation completion rates, technical failures, or exclusions, and whether missing data were adequately addressed; and of 4.5, statistical analysis appropriateness—whether analyses match the research questions and data types; we assessed whether studies used suitable statistical methods for comparing model performance, reported measures of precision (CIs and SEs), and conducted appropriate subgroup or sensitivity analyses. Studies receive “yes” when criteria are clearly met with adequate documentation, “no” when not met, or “cannot tell” when insufficient information prevents assessment [31]. The “cannot tell” rating was assigned when benchmark papers omitted methodological details such as question sampling procedures, validation protocols, interrater reliability statistics, or statistical testing methods, preventing independent verification of quality. Two independent reviewers (EJG and CSB) assessed the methodological quality, and disagreements were resolved through consensus.

### Study Outcomes (Data Items Extracted)

Primary outcome measures included benchmark characteristics (year, version, availability, clinical domain, task type, number of items, and question format), development method (expert involvement and validation methods), psychometric properties: reliability (if reported), validity evidence, and performance metrics (accuracy, *F*_1_-score, area under the curve, and human benchmark scores). Secondary outcomes included target systems (specific LLMs evaluated), geographic scope (countries or regions covered), and implementation (languages supported and computational requirements).

### Synthesis Approach

Given the heterogeneity of benchmark methodologies and evaluation metrics, a narrative synthesis (all 39 included benchmarks) approach was used following established guidance [32]. Due to substantial heterogeneity in evaluation metrics (accuracy, *F*_1_-score, BLEU, ROUGE, and perplexity) and methodologies across benchmarks, quantitative synthesis (meta-analysis) was not feasible [33]. Performance ranges reported (eg, 84%-90% for knowledge benchmarks) represent the actual spectrum of values observed across individual studies, not pooled estimates or converted metrics. Benchmarks were categorized into knowledge-based versus practice-based approaches, with performance metrics reported as originally published for comparative analysis. Special attention was paid to the evolution from static knowledge assessment to dynamic clinical simulation, regulatory implications, and the documented empathy paradox phenomenon.

### Reporting Bias Assessment

We assessed reporting bias by comparing published benchmarks to registered protocols where available, examining selective outcome reporting within studies, and searching for unpublished benchmarks mentioned in included studies. Formal publication bias assessment was not applicable for this review type.

### Certainty Assessment

Given the descriptive nature of this systematic review of benchmarks (not interventions), formal certainty assessment (eg, GRADE [Grading of Recommendations Assessment, Development, and Evaluation]) was not applicable. We instead report confidence in our descriptive findings based on completeness of reporting and methodological quality.

## Results

### Study Selection and Overview

Title and abstract screening was conducted independently by 2 reviewers (EJG and CSB). We piloted the screening criteria on 50 records, achieving substantial agreement (κ=0.82, 95% CI 0.68-0.96). Full-text screening was performed independently with agreement (κ) of 0.89 (95% CI 0.85-0.93). Disagreements were resolved through discussion, with a third reviewer (GHB) consulted for 2 unresolved cases. Reference lists of all included benchmarks and relevant systematic reviews identified during screening were manually examined for additional eligible studies, which yielded 3 additional records through citation searching.

Our systematic review identified 39 medical LLM benchmarks [10,12,16,17,34-68] spanning from 2017 to 2025, representing a comprehensive landscape of evaluation methodologies in clinical medicine. The temporal distribution of these benchmarks reveals an accelerating trend in development, with 23 benchmarks (59%, 95% CI 43.4%-72.9%) published after 2023, suggesting increased recognition of the need for comprehensive LLM evaluation in health care [7]. These benchmarks emerged from 22 countries across 6 continents, with notable contributions from the United States (n=13, 33%), China (n=8, 21%), and emerging participation from previously underrepresented regions including Africa (AfriMed-QA [36] and TRINDs [37]) and Latin America, reflecting growing global recognition of the need for culturally diverse medical AI evaluation. This geographic diversity is particularly important given that medical practices, disease prevalence, and treatment protocols vary significantly across regions.

The collective scope of these benchmarks is substantial: over 2.3 million questions and clinical scenarios across 45 languages spanning 172 medical specialties, with individual benchmarks ranging from highly focused assessments (*JAMA* [*Journal of the American Medical Association*] Clinical Challenge [34]: 308 questions) to comprehensive platforms (MedBench-Platform [35]: 300,901 questions). This 1000-fold variation in scale reflects divergent philosophies about evaluation depth versus breadth, with smaller benchmarks prioritizing carefully curated, expert-validated questions for specific clinical scenarios, while larger benchmarks emphasize comprehensive coverage across medical domains at the potential cost of individual question quality. For instance, JAMA Clinical Challenge’s 308 questions each include detailed clinical vignettes and expert-written explanations, requiring approximately 5-10 minutes per question for human physicians to complete [34]. In contrast, MedBench-Platform’s 300,901 questions provide broad coverage but rely primarily on automated validation, with limited expert review of individual items [35].

**Figure 1:**
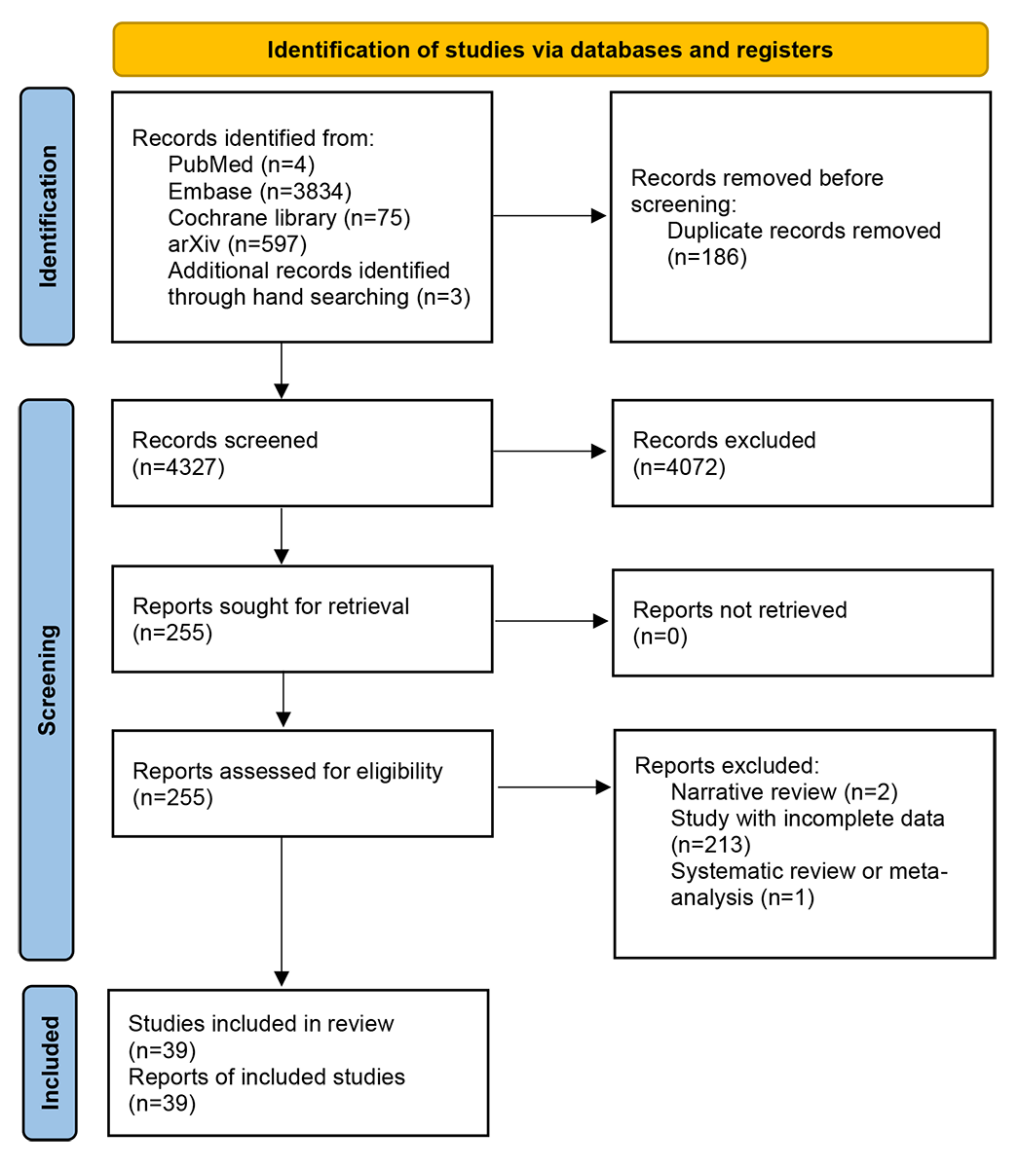
Study selection process.

This heterogeneity in benchmark design reflects the field’s ongoing debate about optimal evaluation strategies: whether to prioritize depth of assessment through carefully validated clinical scenarios or breadth of coverage across the vast landscape of medical knowledge. The coexistence of these divergent approaches suggests that no single evaluation paradigm has emerged as the gold standard for medical LLM assessment. The detailed study selection process is described in Textbox 1 and Figure 1.

The searches were conducted in August 31, 2025. Language limits (English) and publication type limits (Embase: articles, articles in press, and reviews) were applied. Citation searching was performed on all included studies. A comprehensive search strategy was used in this systematic review of medical LLM evaluation benchmarks. Four databases were searched from inception to August 31, 2025: MEDLINE via PubMed (n=4 results), Embase via Ovid (n=3834 results), Cochrane Library (n=75 results), and arXiv preprint repository (n=597 results). Search strategies used controlled vocabulary-informed keywords searched as free-text terms in titles and abstracts ([tiab] for PubMed, :tiab for Embase) targeting three concept domains: (1) LLMs (“large language model,” “LLM,” “GPT,” “ChatGPT,” “artificial intelligence,” and “machine learning”), (2) evaluation frameworks (“benchmark,” “evaluation,” “assessment,” and “performance metric”), and (3) clinical medicine (“medical,” “clinical,” “healthcare,” and “medicine”). Searches were limited to English-language publications. Boolean operators (AND, OR) were used to combine search concepts. The table displays the exact search syntax for each database, including field tags and filters applied, along with the number of records retrieved at each search step.

PRISMA 2020 flow diagram depicting the systematic identification, screening, and selection of medical LLM evaluation benchmarks. The review searched 4 databases (MEDLINE/PubMed, Embase, Cochrane Library, and arXiv) from inception to August 31, 2025, identifying 3917 initial records. After removal of duplicates, 3350 records underwent title and abstract screening by 2 independent reviewers (interrater reliability κ=0.82, 95% CI 0.68-0.96), resulting in 261 reports retrieved for full-text assessment. Full-text screening (κ=0.89, 95% CI 0.85-0.93) excluded 210 reports for the following reasons: wrong study design (n=87), nonbenchmark studies (n=52), nonclinical domains (n=38), insufficient evaluation data (n=21), duplicate benchmarks (n=8), and unavailable full text (n=4). The final systematic review included 39 unique medical LLM benchmarks published between 2017 and 2025, spanning knowledge-based (n=21), practice-based (n=15), and hybrid evaluation approaches (n=3). Two reviewers resolved disagreements through discussion, with a third reviewer consulted for 2 unresolved cases.

### Benchmark Characteristics and Distribution

The identified benchmarks demonstrate substantial heterogeneity in scale, scope, and methodological approaches. Dataset sizes range from 308 questions in specialized assessments such as JAMA Clinical Challenge and Medbullets [34] to over 300,000 questions in comprehensive frameworks such as MedBench-Platform [35]. This variation extends beyond mere numbers to differences in question design, validation methods, and intended use cases.

Geographic representation spans 6 continents, with notable concentrations in North America (13 benchmarks), Asia (12 benchmarks), and Europe (5 benchmarks), while emerging contributions from Africa and Latin America address previously underrepresented medical contexts [36,37]. For example, AfriMed-QA specifically includes questions about diseases endemic to Africa, while TCMBench incorporates Traditional Chinese Medicine concepts that are integral to health care delivery for over a billion people but absent from Western-focused benchmarks [36,58].

Language diversity emerged as an important characteristic, with 18 (46%) benchmarks incorporating non-English content, including specialized medical terminology in Chinese, Korean, Swedish, Spanish, and 15 African languages. This multilingual approach is essential because medical terminology, cultural contexts of illness, and treatment approaches often do not translate directly across languages [6]. This linguistic diversity reflects growing recognition that medical AI must serve global health care needs beyond English-speaking populations [6] (Tables 1 and 2). The 39 benchmarks show diverse validation approaches (Table 1): medical librarian annotation (LiveQA) [38], official examination validation (HEAD-QA) [40], medical doctor expert validation (MedQA) [45], and multistage medical examination validation (MedBench-Edu) [49]. This variation in validation rigor may contribute to the observed performance differences across benchmarks.

Comprehensive summary of 39 medical LLM evaluation benchmarks identified through a systematic review (search period: inception to August 31, 2025). The table presents key characteristics for each benchmark including: study identification (first author, year, and reference number), benchmark name and version, evaluation approach (knowledge-based, practice-based, or hybrid), primary languages assessed, clinical domain coverage (general medicine, specialty-specific, or multispecialty), data source (licensing examinations, real patient records, physician-generated questions, or curated cases), sample size (number of questions, tasks, or conversations), question format (multiple-choice, open-ended, conversational, or multimodal), target models evaluated, geographic origin of development team, and public availability status. Benchmarks span 6 continents with concentration in North America (n=13, 33%), Asia (n=12, 31%), and Europe (n=5, 13%). Total assessment items exceed 2.3 million questions across 45 languages and 172 medical specialties. Temporal analysis reveals 59% (n=23) of benchmarks published after 2023, reflecting rapid evolution in medical AI evaluation.

Quality assessment of 39 medical LLM benchmarks using the MMAT for heterogeneous study designs. Two independent reviewers (CSB and EJG) assessed each benchmark across 7 standardized quality domains with interrater agreement κ=0.91 (95% CI 0.65-1.00, n=5 pilot studies). Assessment criteria include: of S1, clarity of research questions or objectives; of S2, whether collected data adequately address research questions; of 4.1, relevance of sampling strategy to research questions; of 4.2, representativeness of sample for target population; of 4.3, appropriateness of measurements (validity and reliability); of 4.4, risk of nonresponse bias; and of 4.5, appropriateness of statistical analysis for research questions. Each criterion is rated as yes (Y), criterion clearly met with adequate documentation; no (N), criterion not met; or cannot tell (CT), insufficient information reported to make determination. The results reveal substantial methodological heterogeneity: 26% (95% CI 14.6%-41.1%, n=10/39) of benchmarks received predominantly “cannot tell” ratings across multiple domains, indicating incomplete methodological reporting. Common gaps include undocumented sampling strategies, unclear validation procedures, and absent statistical considerations. High-quality benchmarks (eg, MedQA, HealthBench, and MedAgentBench) demonstrate comprehensive methodology reporting across all assessed domains. Quality assessment were conducted between September and October 2025 following PRISMA guidelines for systematic reviews.

**Table 1:** Clinical summary of the included studies.

Study and year	Benchmark	Type	Language of LLM^a^	Dataset	Question source	Validation
Abacha et al, 2017 [38]	LiveQA	Knowledge	English	634 pairs of medical questions and answers	National Library of Medicine consumer health questions	Medical librarian annotation
Abacha et al, 2019 [39]	MedicationQA	Knowledge	English	674 pairs of medical questions and answers	Real consumer questions	Medical experts, including doctors
Vilares et al, 2019 [40]	HEAD-QA	Knowledge	Spanish	6765 questions, including 1149 medical questions	Spanish health care licensing examinations	Official examination validation
Jin et al, 2019 [41]	PubMedQA	Hybrid	English	211.3K artificially generated questions and answers	PubMed abstracts	Expert annotation
Zeng et al, 2020 [42]	MedDialog	Practice	English or Chinese	3.4 million Chinese and 0.26 million English conversations	Online medical consultations	Basic data cleaning without clinical validation
Hendrycks et al, 2021 [43]	MMLU^b^ Medical	Knowledge	English	1785 medical questions	Academic or professional examinations	Academic validation without clinical review
Liu et al, 2022 [44]	MedDG	Practice	Chinese	17,864 Chinese dialogues	Online medical consultation platform	Domain expert annotation
Jin et al, 2021 [45]	MedQA	Knowledge	English or Chinese	61,097 medical and clinical questions	USMLE^c^, MCMLE^d^, and TWMLE^e^	MD^f^ expert validation
Yan et al, 2022 [46]	ReMeDi	Practice	Multilanguage	96,965 conversations	Multidomain medical dialogues	Fine-grained medical labels
Pal et al, 2022 [47]	MedMCQA	Knowledge	English	194,000+ questions	AIIMS^g^/NEET PG^h^ examinations	Authentic examination curation
Liu et al, 2023 [48]	CMExam	Knowledge	Chinese	68,119 questions	Chinese National Medical Licensing Examination	GPT-4 assisted human verification of apnea measures
Singhal et al, 2023 [12]	HealthSearchQA	Knowledge	English	3173 commonly searched consumer medical questions	Search engine queries	Consumer validation
Cai et al, 2024 [49]	MedBench-Edu	Knowledge	Chinese	40,041 questions	Chinese Medical Licensing Examination, Resident Standardization Training Examination, Doctor In-Charge Qualification Examination, and real-world clinic cases	4-stage medical examination validation
Chen et al, 2025 [34]	JAMA^i^ Clinical Challenge and Medbullets	Practice	English	308 USMLE Step 2&3 style questions (Medbullets), 1524 clinical cases collected from the JAMA Network Clinical Challenge archive (JAMA Clinical Challenge)	USMLE Step 2&3, JAMA	Expert-written explanations
Kim et al, 2024 [50]	MedExQA	Knowledge	English	965 questions	5 medical specialties	Multiple expert explanations
Liu et al, 2024 [35]	MedBench-Platform	Knowledge	Chinese	300,901 questions	Chinese medical examinations	Standardized cloud-based evaluation
Hertzberg et al, 2024 [51]	MedQA-SWE	Practice	Swedish	3180 questions	Swedish medical licensing examination	Manual curation
Kweon et al, 2024 [52]	KorMedMCQA	Knowledge	Korean	7469 questions	Korean health care licensing examinations	Chain-of-thought reasoning assessment
Schmidgall et al, 2024 [53]	BiasMedQA	Knowledge	English	1273 questions	MedQA with bias prompts	Cognitive bias validation
Rawat et al, 2024 [54]	DiversityMedQA	Knowledge	English	2109 questions	MedQA with demographic perturbations	GPT-4 filtered validation
Liu et al, 2024 [55]	ClinicBench	Practice	English	17 datasets and 11 tasks	Clinical scenarios	Medical expert evaluation
Ouyang et al, 2024 [56]	CliMedBench	Practice	Chinese	33,735 questions	Real-world medical reports	Expert-guided construction
Yao et al, 2024 [57]	MedQA-CS	Practice	English	1667 (instruction, input, and output) data points	OSCE^j^-inspired scenarios	AI-SCE^k^ framework
Yue et al, 2024 [58]	TCMBench	Knowledge	Chinese	5473 questions	Traditional Chinese Medicine Licensing Exam	Traditional Chinese Medicine Licensing Exam score evaluation
Zhu et al, 2025 [59]	DentalBench	Knowledge	Bilingual	36,597 questions	16 dental subfields	Expert curation
Zhang et al, 2025 [60]	LLMEval-Med	Practice	Multilanguage	2996 questions	Real-world electronic health records and expert-designed clinical scenarios	Physician validation
Olantunji et al, 2025 [36]	AfriMed-QA	Knowledge	English	15,275 questions	Pan-African medical schools	621 contributors
Fan et al, 2024 [61]	Multi-View Medical Evaluation (MVME) benchmark	Practice	Chinese	506 high-quality case records	Website that compiles an extensive database of clinical cases in Chinese	Multiagent simulation
Huang et al, 2024 [62]	ChatCoach	Practice	English	3500 conversations with 13,666 utterances	99 diseases from real-world medical consultations (MedDialog dataset) + synthetic coaching	2-3 annotators (either medical professionals or knowledgeable students) + GPT-4
Abacha et al, 2025 [63]	MEDEC	Practice	English	3848 clinical texts	US hospital systems	Medical annotator validation
Zuo et al, 2025 [64]	MedXpertQA	Knowledge	English	4460 questions (2455 text + 2005 multimodal)	USMLE, COMLEX-USA^l^ for general, 17 American specialty board examinations for specialized scenarios, and 3 image-rich sources, such as the NEJM^m^ Image Challenges	Licensed physicians, multiple rounds
Asiedu et al, 2025 [37]	TRINDs	Knowledge	Multilanguage	11,719 prompts	Authoritative medical sources	7 experts (quality assessment)
Arora et al, 2025 [16]	HealthBench	Practice	Multilanguage	5000 multiturn conversations between a model and an individual user or health care professional	Physician-created scenarios	262 physicians, 60 countries
Jiang et al, 2025 [65]	MedAgentBench	Practice	English	300 patient-specific clinically derived tasks	Stanford Hospital records	Licensed physician curation
Wang et al, 2025 [17]	CSEDB^n^	Practice	English	2069 open-ended questions	26 clinical departments and 30 assessment criteria, including 17 safety-focused and 13 effectiveness-focused indicators	32 specialist physicians
Bedi et al, 2025 [10]	MedHELM^o^	Hybrid	English	35 benchmarks (17 existing, 18 newly formulated)	121 clinical tasks	29 clinician validation
Lamparth et al, 2025 [66]	MENTAT	Practice	English	203 questions	Mental health scenarios	Clinician annotation
Avnat et al, 2025 [67]	EBMQA^p^	Hybrid	English	105,222 questions	50,000 peer-reviewed publications and more than 20,000,000 medical relations	Medical expert validation
Zhu et al, 2025 [68]	DiagnosisArena	Practice	English	1113 cases	10 top-tier medical journals	Expert screening review

^a^LLM: large language model.

^b^MMLU: Massive Multitask Language Understanding.

^c^USMLE: United States Medical Licensing Examination.

^d^MCMLE: Mainland China Medical Licensing Examination.

^e^TWMLE: Taiwan Medical Licensing Examination.

^f^MD: medical doctor.

^g^AIIMS: All India Institute of Medical Sciences.

^h^NEET PG: National Eligibility cum Entrance Test for Postgraduate.

^i^JAMA: Journal of the American Medical Association.

^j^OSCE: objective structured clinical examination.

^k^AI-SCE: Artificial Intelligence–Structured Clinical Examination.

^l^COMLEX-USA: Comprehensive Osteopathic Medical Licensing Examination of the United States.

^m^NEJM: New England Journal of Medicine.

^n^CSEDB: Clinical Safety-Effectiveness Dual-Track Benchmark.

^o^MedHELM: Medical Holistic Evaluation of Language Models.

^p^EBMQA: Evidence-Based Medicine Question Answering.

**Table 2:** Quality assessment of medical LLM^a^ benchmarks (MMAT^b^ criteria).

Study and year	Benchmark	S1^c^	S2^d^	4.1^e^	4.2^f^	4.3^g^	4.4^h^	4.5^i^
Abacha et al, 2017 [38]	LiveQA	Y^j^	Y	Y	CT^k^	Y	CT	Y
Abacha et al, 2019 [39]	MedicationQA	Y	Y	Y	CT	Y	CT	Y
Vilares et al, 2019 [40]	HEAD-QA	Y	Y	Y	Y	Y	Y	Y
Jin et al, 2019 [41]	PubMedQA	Y	Y	Y	Y	Y	Y	Y
Zeng et al, 2020 [42]	MedDialog	Y	Y	Y	Y	Y	Y	Y
Hendrycks et al, 2021 [43]	MMLU^l^ Medical	Y	Y	Y	Y	Y	Y	Y
Liu et al, 2022 [44]	MedDG	Y	Y	Y	CT	Y	CT	Y
Jin et al, 2021 [45]	MedQA	Y	Y	Y	Y	Y	Y	Y
Yan et al, 2022 [46]	ReMeDi	Y	Y	Y	Y	Y	Y	Y
Pal et al, 2022 [47]	MedMCQA	Y	Y	Y	Y	Y	Y	Y
Liu et al, 2023 [48]	CMExam	Y	Y	Y	Y	Y	Y	Y
Singhal et al, 2023 [12]	HealthSearchQA	Y	Y	Y	CT	Y	CT	Y
Cai et al, 2024 [49]	MedBench-Edu	CT	CT	CT	CT	CT	CT	CT
Chen et al, 2025 [34]	JAMA^m^ Clinical Challenge and Medbullets	Y	Y	Y	Y	Y	Y	Y
Kim et al, 2024 [50]	MedExQA	Y	Y	Y	Y	Y	Y	Y
Liu et al, 2024 [35]	MedBench-Platform	CT	CT	CT	CT	CT	CT	CT
Hertzberg et al, 2024 [51]	MedQA-SWE	Y	Y	Y	Y	Y	Y	Y
Kweon et al, 2024 [52]	KorMedMCQA	Y	Y	Y	Y	Y	Y	Y
Schmidgall et al, 2024 [53]	BiasMedQA	Y	Y	Y	Y	Y	Y	Y
Rawat et al, 2024 [54]	DiversityMedQA	Y	Y	Y	Y	Y	Y	Y
Liu et al, 2024 [55]	ClinicBench	Y	Y	Y	Y	Y	Y	Y
Ouyang et al, 2024 [56]	CliMedBench	Y	Y	Y	Y	Y	Y	Y
Yao et al, 2024 [57]	MedQA-CS	Y	Y	Y	Y	Y	Y	Y
Yue et al, 2024 [58]	TCMBench	Y	Y	Y	Y	Y	Y	Y
Zhu et al, 2025 [59]	DentalBench	Y	Y	Y	Y	Y	Y	Y
Zhang et al, 2025 [60]	LLMEval-Med	Y	Y	Y	Y	Y	Y	Y
Olantunji et al, 2025 [36]	AfriMed-QA	Y	Y	Y	Y	Y	Y	Y
Fan et al, 2024 [61]	Multi-View Medical Evaluation (MVME) benchmark	CT	CT	CT	CT	CT	CT	CT
Huang et al, 2024 [62]	ChatCoach	CT	CT	CT	CT	CT	CT	CT
Abacha et al, 2025 [63]	MEDEC	Y	Y	Y	Y	Y	Y	Y
Zuo et al, 2025 [64]	MedXpertQA	Y	Y	Y	Y	Y	Y	Y
Asiedu et al, 2025 [37]	TRINDs	CT	CT	CT	CT	CT	CT	CT
Arora et al, 2025 [16]	HealthBench	Y	Y	Y	Y	Y	Y	Y
Jiang et al, 2025 [65]	MedAgentBench	Y	Y	Y	Y	Y	Y	Y
Wang et al, 2025 [17]	CSEDB^n^	Y	Y	Y	Y	Y	Y	Y
Bedi et al, 2025 [10]	MedHELM^o^	Y	Y	Y	Y	Y	Y	Y
Lamparth et al, 2025 [66]	MENTAT	CT	CT	CT	CT	CT	CT	CT
Avnat et al, 2025 [67]	EBMQA^p^	CT	CT	CT	CT	CT	CT	CT
Zhu et al, 2025 [68]	DiagnosisArena	CT	CT	CT	CT	CT	CT	CT

^a^LLM: large language model.

^b^MMAT: Mixed Methods Appraisal Tool.

^c^S1: clarity of research questions or objectives.

^d^S2: whether collected data adequately address research questions.

^e^4.1: relevance of sampling strategy to research questions.

^f^4.2: representativeness of sample for target population.

^g^4.3: appropriateness of measurements (validity and reliability).

^h^4.4: risk of nonresponse bias.

^i^4.5: appropriateness of statistical analysis for research questions.

^j^Y: yes, criterion clearly met with adequate documentation.

^k^CT: cannot tell, insufficient information reported to make determination.

^l^MMLU: Massive Multitask Language Understanding.

^m^JAMA: Journal of the American Medical Association.

^n^CSEDB: Clinical Safety-Effectiveness Dual-Track Benchmark.

^o^MedHELM: Medical Holistic Evaluation of Language Models.

^p^EBMQA: Evidence-Based Medicine Question Answering.

### Classification and Distribution of Benchmark Types

Analysis revealed 3 primary benchmark categories with distinct evaluation paradigms. Knowledge-based benchmarks comprise 21 datasets (54%, 95% CI 38.6%-68.4%), focusing on medical factual recall and standardized examination performance. Practice-based benchmarks account for 15 datasets (38%, 95% CI 24.9%-54.1%), emphasizing clinical reasoning, workflow integration, and real-world decision-making scenarios. Hybrid approaches represent 3 benchmarks (8%, 95% CI 2.7%-20.3%), combining knowledge assessment with practical application evaluation. This distribution reflects an evolving recognition that medical competency extends beyond factual knowledge to encompass clinical judgment, communication skills, and contextual reasoning [23].

### Knowledge-Based Benchmark Analysis

Knowledge-based benchmarks predominantly derive from professional licensing examinations, with MedQA [45] using 61,097 USMLE-style questions across 3 languages and MedMCQA [47] incorporating 194,000+ questions from Indian postgraduate entrance examinations. These benchmarks represent decades of accumulated examination content, with each question having been validated through actual use in high-stakes medical testing.

The regional variations in these benchmarks reveal important differences in medical education and practice. The HEAD-QA benchmark [40] from Spain contributes 6765 questions from health care licensing examinations, including content specific to the European health care context. CMExam [48] provides 68,119 questions from Chinese National Medical Licensing Examinations, notably incorporating Traditional Chinese Medicine alongside Western medicine with demonstrating global standardization efforts in medical knowledge assessment. KorMedMCQA [52] adds 7469 questions from Korean health care licensing examinations, reflecting Korea’s specific disease epidemiology and health care system.

These benchmarks achieved the highest model performance metrics, with GPT-4 (OpenAI) and Claude-3 Opus (ANTHROPIC PBC) consistently exceeding 85% accuracy on multiple-choice formats [1,12]. However, this high performance on multiple-choice questions may not translate to clinical competence, as the format allows for educated guessing and pattern recognition rather than requiring the generation of diagnostic reasoning or treatment plans from scratch [11]. The multiple-choice format inherently simplifies complex medical scenarios, potentially overestimating actual clinical competence [11]. Furthermore, these benchmarks exhibit limited assessment of uncertainty handling, with only 3 of 18 knowledge-based benchmarks incorporating questions where “insufficient information” represents a valid response.

### Practice-Based Benchmark Evolution

Practice-based benchmarks demonstrate markedly different characteristics and performance patterns, representing a shift in evaluation philosophy. These benchmarks attempt to capture the complexity of real clinical practice, where physicians must integrate incomplete information, manage uncertainty, and make time-sensitive decisions.

MedAgentBench [65] introduces 300 clinically derived tasks within a Fast Healthcare Interoperability Resources (FHIR)–compliant virtual electronic health records (EHRs) environment containing 700,000+ data elements from Stanford Hospital records. This represents one of the most realistic clinical environments created for LLM evaluation, requiring models to navigate the same fragmented, incomplete, and sometimes contradictory information that physicians encounter in actual EHR systems. This benchmark requires models to navigate authentic patient records, synthesize longitudinal data across multiple encounters, and make time-sensitive decisions while managing incomplete information. The sophistication of this approach reveals that even frontier models achieve only 69.67% success rate when required to integrate multiple information sources and maintain clinical context over extended interactions.

HealthBench [16] uses 5000 physician-validated multiturn conversations assessed through custom rubrics created by 262 clinicians across 60 countries. The involvement of clinicians from 60 countries ensures that the benchmark captures diverse clinical practices and cultural contexts, addressing a limitation of US-centric evaluations. Each conversation undergoes evaluation across 12 distinct clinical competencies including history-taking, physical examination interpretation, differential diagnosis formulation, treatment planning, and shared decision-making. The multiturn nature of these conversations is crucial, as real clinical encounters involve iterative information gathering and refinement of diagnostic hypotheses rather than single-shot decisions.

The CSEDB [17], developed by 32 specialist physicians across 26 clinical departments, evaluates both safety and effectiveness through 2069 open-ended questions. The framework uses 17 safety-focused indicators assessing harm prevention, contraindication recognition, and risk communication, alongside 13 effectiveness-focused metrics evaluating diagnostic accuracy, treatment appropriateness, and clinical efficiency. The separation of safety and effectiveness metrics is particularly important, as a treatment may be effective but unsafe in certain contexts, or safe but suboptimal—distinctions that are important in clinical practice. Maximum achievable scores of 0.912 for safety and 0.861 for effectiveness reflect the inherent complexity of clinical practice where perfect performance remains elusive even for experienced physicians.

ClinicBench [55] presents 17 datasets encompassing 11 distinct clinical tasks, ranging from admission note generation to discharge planning. The benchmark’s comprehensive scope reveals task-specific performance variations, with models achieving 75% accuracy on structured tasks such as laboratory interpretation but only 45% success on complex activities requiring integration of psychosocial factors into clinical decision-making.

CliMedBench [56], developed from Chinese tertiary hospital data, contributes 33,735 questions derived from real-world medical reports across 14 clinical scenarios. The benchmark’s unique strength lies in its inclusion of actual clinical documentation, exposing models to the abbreviated terminology, incomplete sentences, and contextual references characteristic of authentic medical records.

MedQA-CS [57] introduces an AI-SCE (Structured Clinical Examination) framework with 1667 objective structured clinical examination–inspired scenarios evaluating clinical skills beyond knowledge recall. The benchmark assesses procedural competence, patient interaction capabilities, and clinical reasoning through structured clinical vignettes requiring sequential decision-making.

MEDEC [63] focuses on error detection and correction within 3848 clinical texts from US hospital systems, addressing the significant but understudied capability of identifying and correcting medical documentation errors. The benchmark reveals that models miss 38% of clinically significant errors readily identified by human physicians, highlighting gaps in practical clinical review capabilities.

DiagnosisArena [68] uses 1113 complex diagnostic cases from top-tier medical journals including *NEJM* (*New England Journal of Medicine*), *Lancet*, and *JAMA*. The benchmark’s paired case design, where each diagnosis has a similar-presenting alternative, exposes models’ inability to distinguish subtle clinical nuances. Even advanced reasoning models achieve only 45.82% (95% CI 42.9%-48.8%) accuracy, while practicing physicians average 20% on identical cases, suggesting these represent genuinely challenging clinical scenarios.

Additional practice benchmarks include MedDialog [42] with 3.4 million medical conversations, MedDG [44] offering 17,864 Chinese dialogues, ReMeDi [46] encompassing 96,965 conversations across 843 diseases, LLMEval-Med [60] with 2996 EHR-derived questions, ChatCoach [62] evaluating therapeutic communication, and MENTAT [66] focusing on mental health care scenarios.

### Hybrid Benchmark Approaches

Hybrid benchmarks represent sophisticated attempts to bridge the gap between knowledge assessment and practice evaluation, recognizing that clinical competence requires seamless integration of theoretical understanding with practical application [6,23]. MedHELM (Medical Holistic Evaluation of Language Models) [10] stands as the most comprehensive hybrid framework, integrating 35 subbenchmarks that span 121 distinct clinical tasks validated by 29 clinicians. This framework uniquely combines traditional multiple-choice questions with open-ended clinical scenarios, revealing that models exhibiting strong performance on isolated knowledge questions often fail when the same knowledge requires contextual integration within patient care workflows. MedHELM’s multidimensional assessment demonstrates that medical competence cannot be adequately measured through singular evaluation paradigms.

PubMedQA [41] operates at the intersection of knowledge retrieval and evidence synthesis, using 211,300 artificially generated questions alongside 1000 expert-labeled instances derived from PubMed abstracts. The benchmark assesses not only the ability to extract factual information from medical literature but also the capacity to evaluate research quality, recognize study limitations, and synthesize findings into clinically actionable recommendations. This dual focus reveals important gaps in models’ abilities to transition from understanding individual research findings to applying evidence-based medicine principles in clinical decision-making.

EBMQA (Evidence-Based Medicine Question Answering) [67] further advances hybrid evaluation by incorporating 105,222 questions derived from over 50,000 peer-reviewed publications and 20 million medical relations. The benchmark uniquely evaluates models’ capacity to navigate the hierarchy of evidence, assess methodological rigor, and appropriately weight conflicting research findings. Performance analysis reveals systematic difficulties in distinguishing between correlation and causation, evaluating external validity, and recognizing publication bias—skills essential for evidence-based clinical practice. These hybrid approaches collectively demonstrate that effective medical AI evaluation requires multifaceted assessment methodologies that neither pure knowledge nor pure practice benchmarks can provide independently [7,18,69].

### Temporal Trends in Benchmark Development

**Figure 2:**
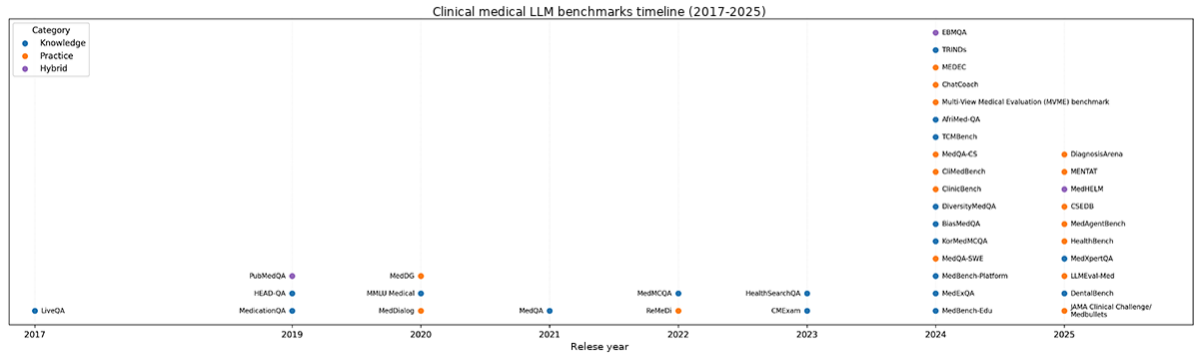
Timeline of clinical medical large language model benchmarks. Temporal distribution of 39 medical LLM benchmarks from 2017 to 2025. Each point represents a benchmark publication, illustrating the acceleration of benchmark development after 2023 and the evolution from knowledge-based to practice-based evaluation approaches. LLM: large language model.

Longitudinal analysis reveals clear evolutionary patterns in benchmark sophistication (Figure 2). Early benchmarks (2017-2020) focused primarily on single-modality text processing and factual retrieval, exemplified by LiveQA Medical’s [38] 634 consumer health questions and MedicationQA’s [39] 674 medication-related queries. These pioneering efforts established baseline evaluation methodologies but remained constrained by limited scope and simplified question formats.

The intermediate period (2021-2023) witnessed expansion into multimodal assessment and specialized domains. MedQA’s [45] introduction of 61,097 questions from multiple international licensing examinations marked a significant scale increase, while ReMeDi’s [46] 96,965 multidomain conversations introduced dialogue-based evaluation. Specialized benchmarks emerged during this period, with TCMBench [58] addressing Traditional Chinese Medicine through 5473 questions and CMExam [48] incorporating 68,119 questions from Chinese licensing examinations, reflecting growing recognition of diverse medical traditions and regional practices.

The current generation (2024-2025) emphasizes ecological validity through simulated clinical environments and real-world data integration. AI Hospital’s [61] MVME multiagent framework, MedAgentBench’s [65] FHIR-compliant EHR environment, and DiagnosisArena’s [68] use of complex journal cases represent shifts toward practice-oriented evaluation. This evolution reflects maturation in understanding that medical AI evaluation requires assessment methodologies that mirror clinical practice complexity rather than simplified knowledge testing [18].

Timeline depicting the evolution of 39 medical LLM evaluation benchmarks from 2017 to 2025 identified through a systematic review (search date: August 31, 2025). Each data point represents a unique benchmark publication positioned by publication year and categorized by evaluation approach: knowledge-based (green circles, n=21, 54%), practice-based (blue squares, n=15, 38%), and hybrid frameworks (red triangles, n=3, 8%). The visualization reveals three distinct developmental phases: (1) foundational phase (2017-2020, n=6 benchmarks): early frameworks establishing baseline evaluation through factual knowledge assessment (eg, LiveQA, MedicationQA, and HEAD-QA); (2) expansion phase (2021-2023, n=10 benchmarks): increasing sophistication with multimodal assessment, specialized domains, and larger-scale datasets (eg, MedQA 61,097 questions, MedMCQA 194,000+ questions); and (3) practice-oriented phase (2024-2025, n=23 benchmarks): rapid acceleration with 59% (95% CI 43.4%-72.9%) of all benchmarks published in this period, emphasizing ecological validity through simulated clinical environments, real EHR integration, and assessment of safety or effectiveness in authentic workflows (eg, HealthBench 5000 conversations, MedAgentBench 300 EHR tasks, and CSEDB 2069 safety scenarios). The acceleration after 2023 (23 benchmarks in 2 years versus 16 benchmarks in 6 prior years) reflects growing recognition that medical licensing examination performance inadequately predicts clinical competence. Geographic distribution spans 6 continents with contributions from academic institutions, health care systems, and AI research laboratories worldwide.

### Performance Patterns Across Benchmark Categories

Systematic performance analysis reveals variations between knowledge demonstration and clinical application [7]. On knowledge-based benchmarks, frontier models achieve 84%-90% accuracy, approaching or exceeding average physician performance on standardized examinations [1,12]. This represents remarkable progress from early models that achieved <60% on the same benchmarks just 2 years prior. However, performance on practice-based benchmarks varies considerably, with DiagnosisArena reports 45.82% accuracy for advanced models on complex diagnostic cases [68], MedAgentBench shows 69.67% (95% CI 64.2%-74.6%) success rate for the best-performing model in EHR navigation tasks [65], and HealthBench demonstrates approximately 60% (95% CI 58.6%-61.3%) performance on realistic clinical conversations [16]. The distinction between knowledge and practice benchmarks remains poorly standardized in the literature, but these results consistently show lower performance on practice-based assessments [6,7].

Task-specific analysis reveals differential performance patterns across evaluation categories [1,10,16]. Factual retrieval tasks maintain relatively high performance at 85%-93% [45], while clinical reasoning tasks show moderate degradation to 50%-60% accuracy [55,65]. Diagnostic tasks exhibit more severe challenges, with models achieving 45%-55% success rates on complex cases [68]. Safety assessment tasks revealed performance limitations in our review, with benchmarks showing 40%-50% accuracy despite the life-critical nature of these capabilities [17] (Table 3). These task-specific variations suggest that different cognitive capabilities required for medical practice are not uniformly developed in current LLMs, with particular weaknesses in areas requiring integration, synthesis, and safety assessment—all critical for patient care.

Table 4 presents detailed performance comparisons for 15 major benchmarks, revealing systematic performance degradation across question formats: open-ended diagnostic tasks (DiagnosisArena: 45.82%, 95% CI 42.9%-48.8%) [68] demonstrated markedly lower accuracy than multiple-choice knowledge assessment (MedQA: 78.6%, 95% CI 76.3%-80.9%) [45], with CI widths reflecting both sample size differences and task heterogeneity inherent to practice-based evaluation.

Systematic comparison of LLM performance across 39 medical benchmarks categorized by evaluation approach and clinical task type, revealing a significant knowledge-practice performance gap. Knowledge-based benchmarks (n=21, 54%) assessing factual medical knowledge through multiple-choice questions demonstrate an 84%-90% accuracy range, with frontier models (GPT-4 and Claude-3 Opus) approaching or exceeding average physician performance on licensing examinations. Practice-based benchmarks (n=15, 38%) evaluating real-world clinical competence show substantially lower performance: 45%-69% accuracy range across tasks requiring clinical reasoning, diagnostic synthesis, and workflow integration. Specific examples include DiagnosisArena 45.82% (95% CI 42.9%-48.8%, n=1113 complex cases), MedAgentBench 69.67% (95% CI 64.2%-74.6%, n=300 EHR tasks), and HealthBench 60% (95% CI 58.6%-61.3%, n=5000 conversations). Task-specific performance analysis reveals differential degradation patterns: factual retrieval maintains 85%-93% accuracy; clinical reasoning drops to 50%-60%; diagnostic tasks decline to 45%-55%; and safety-critical scenarios show concerning 40%-50% accuracy despite life-threatening implications. Performance data extracted from original benchmark publications (2017-2025) represent best-performing models at the time of publication, with variations reflecting differences in task complexity, evaluation methodology, and model architecture. The 39-45 percentage point gap between knowledge demonstration and clinical practice performance underscores limitations in current evaluation paradigms and highlights the necessity for practice-based validation before clinical deployment. All percentages represent point estimates; CIs are provided for individual benchmarks where sample sizes permit calculation.

**Table 3:** Comparative performance summary across benchmark types.

Benchmark category		Participants, N	Accuracy	Representative examples	Key assessment focus
Knowledge-based benchmarks		21	84%-90%	MedQA [45], MedMCQA [47], CMExam [48], HealthSearchQA [12]	Medical factual recall, multiple choice questions, and answer performance, licensing examination questions
Practice-based benchmarks		15	45%-69%	DiagnosisArena [68], MedAgentBench [65], HealthBench [16]	Clinical scenarios, real-world decisions
**Task-specific performance**					
	Factual retrieval	1	85%-93%	MedQA [45]	Information extraction
	Clinical reasoning	2	50%-60%	ClinicBench [55], CliMedBench [56]	Treatment planning
	Diagnostic tasks	1	45%-55%	DiagnosisArena [68]	Differential diagnosis
	Safety assessment	1	40-50	CSEDB [17]	Harm prevention
	Hybrid benchmarks	3	Not reported as a single metric	MedHELM [10], PubMedQA [41], EBMQA [67]	Knowledge and application

**Table 4:** Detailed performance comparison of major medical LLM^a^ benchmarks by evaluation type.

Benchmark category			Question format		Metric		Best model performance
**Knowledge-based benchmarks**							
	MedQA [45]	MCQ^b^ (4-choice)		Accuracy		78.6% (95% CI 76.3%-80.9%; GPT-4)	
	MedMCQA [47]	MCQ (4-choice)		Accuracy		72.4% (95% CI 71.3%-73.5%; GPT-4)	
	CMExam [48]	MCQ		Accuracy		61%-71%	
	HEAD-QA [40]	MCQ (4-choice)		Accuracy		69.2%	
	HealthSearchQA [12]	Open-ended		ROUGE-L^c^		0.428	
**Practice-based benchmarks**							
	DiagnosisArena [68]	Open-ended diagnosis		Top-1 accuracy		45.82% (95% CI 42.9%-48.8%; o3-mini)	
	MedAgentBench [65]	EHR^d^ task completion		Success rate		69.67% (95% CI 64.2%-74.6%; Claude-3.5)	
	HealthBench [16]	Multiturn conversations		Rubric score		60% (95% CI 58.6%-61.3%; o3)	
	CSEDB [17]	Open-ended clinical		Accuracy		54.7% (95% CI 53.6%-55.8%; average)	
	ClinicBench [55]	11 clinical tasks		Task-specific		45%-75%	
	CliMedBench [56]	Real clinical reports		Accuracy		50%-60%	
	MEDEC [63]	Error detection		Error detection		62%	
**Hybrid benchmark**							
	MedHELM [10]	Mixed formats		Composite		Varies by task	
	PubMedQA [41]	Evidence synthesis		Accuracy		60%-70%	
	EBMQA [67]	Evidence-based QA^e^		Accuracy		Variable	

^a^LLM: large language model.

^b^MCQ: multiple choice question.

^c^ROUGE-L: Recall-Oriented Understudy for Gisting Evaluation-Longest Common Subsequence.

^d^EHR: electronic health record.

^e^QA: question and answer.

Detailed individual benchmark comparisons (Table 4) further illustrate the knowledge-practice performance gap across specific evaluation frameworks. Knowledge-based benchmarks consistently achieved 70%-79% mean accuracy using multiple-choice formats (MedQA [45]: 78.6%, 95% CI 76.3%-80.9%; MedMCQA [47]: 72.4%, 95% CI 71.3%-73.5%; HEAD-QA [40]: 69.2%), while practice-based assessments using open-ended clinical tasks demonstrated substantially lower performance (DiagnosisArena [68]: 45.82%, 95% CI 42.9%-48.8%; MedAgentBench [65]: 69.67%, 95% CI 64.2%-74.6%; and HealthBench [16]: 60%, 95% CI 58.6%-61.3%). Format dependency emerges as an important determinant: even when assessing similar clinical domains, open-ended formats requiring free-text diagnostic reasoning yielded accuracy 30-40 percentage points lower than multiple-choice factual recall. Statistical precision varied inversely with task complexity, with practice-based benchmarks showing wider CIs (MedAgentBench [65] CI width: 10.4 percentage points) compared to large-scale knowledge assessments (MedQA [45] CI width: 4.6 percentage points), reflecting both smaller sample sizes and inherent heterogeneity in clinical decision-making tasks.

Performance metrics were for 15 representative benchmarks: knowledge-based (n=5), practice-based (n=7), and hybrid (n=3). Table 4 shows question format, primary performance metric, best model performance, and 95% CIs (calculated using the Wilson score method, where sample sizes permitted). Knowledge-based benchmarks demonstrate 70%-79% mean accuracy, while practice-based benchmarks show 46%-70% mean performance, revealing a substantial knowledge-practice gap. Format dependency is evident: open-ended tasks (DiagnosisArena: 45.82%) show markedly lower performance than multiple-choice question formats (MedQA: 78.6%). Performance metrics vary by measurement type (accuracy, ROUGE-L [Recall-Oriented Understudy for Gisting Evaluation-Longest Common Subsequence], and rubric scores) and are not directly comparable. CSEDB safety or effectiveness scores represent the average across 6 models; domain-specific models (MedGPT: 91.2%) outperformed general-purpose LLMs. Benchmarks were selected based on MMAT quality assessment, sample size, and clinical relevance. Detailed characteristics are available in Tables 1 and 2.

### Dataset Availability and Reproducibility

Medical LLM benchmark accessibility varies considerably across different platforms and formats. Several benchmarks demonstrate exemplary accessibility through established data-sharing platforms. DiversityMedQA maintains its dataset on Hugging Face [54], providing researchers with standardized access protocols and version control. Similarly, MedAgentBench offers comprehensive documentation through its GitHub (GitHub, Inc) repository, including detailed FHIR specifications [65] that enable systematic integration with health care systems and facilitate reproducible research practices.

Documentation quality represents another important dimension of reproducibility. HealthBench exemplifies rigorous documentation standards through its detailed rubric documentation, encompassing 48,562 unique evaluation criteria developed collaboratively by 262 physicians representing 60 countries [16]. This extensive international collaboration ensures comprehensive coverage of medical knowledge domains while maintaining clinical relevance across diverse health care contexts.

However, accessibility challenges persist across the benchmark landscape. Many benchmarks lack standardized distribution mechanisms or comprehensive documentation, creating barriers to reproducible research. The absence of unified access protocols often requires researchers to navigate varying data formats, licensing agreements, and technical specifications [1,10].

### Quality Assessment

Quality assessment of medical LLM benchmarks requires systematic evaluation across multiple methodological dimensions. The MMAT provides a structured framework for evaluating benchmark quality through standardized criteria covering methodological rigor, documentation completeness, and research design appropriateness [31]. This assessment approach enables researchers to identify benchmarks with robust methodological foundations while recognizing areas requiring improvement.

Several benchmarks demonstrate exemplary methodological documentation and design principles. High-quality benchmarks such as MedQA [45], HealthBench [16], and MedAgentBench [65] typically provide comprehensive descriptions of data collection procedures, validation methodologies, and evaluation frameworks. Conversely, some benchmarks present challenges in quality assessment due to incomplete methodological reporting or unclear evaluation criteria (Table 1).

Notably, 10 benchmarks (26%, 95% CI 14.6%-41.1%) received predominantly “cannot tell” ratings across MMAT criteria (Table 2). This high proportion of unclear ratings indicates substantial limitations in methodological reporting within the medical LLM benchmark literature. The inability to assess basic quality criteria such as sampling strategy relevance, sample representativeness, and statistical analysis appropriateness suggests that many benchmark papers prioritize technical innovation over rigorous methodological documentation. This finding reveals an important gap: while these benchmarks may contain valuable datasets, insufficient reporting limits their reproducibility and validity assessment.

### Reporting Bias Assessment

Assessment of potential reporting bias was conducted through multiple approaches. We searched for registered protocols or preregistration documentation for the included benchmarks; however, systematic protocol registration was not the standard practice in this field. We examined included studies for evidence of selective outcome reporting by comparing stated objectives with reported results; no clear instances of selective reporting were identified. Gray literature searches and examination of citations within included studies did not reveal unpublished benchmarks meeting our inclusion criteria. The overall risk of reporting bias from missing benchmarks was assessed as low.

### Certainty of Descriptive Findings

Given the descriptive nature of this systematic review examining benchmark characteristics rather than intervention effects, traditional certainty assessment frameworks (eg, GRADE) were not applicable. Instead, we assessed confidence in our descriptive findings based on search comprehensiveness and reporting quality. We have high certainty in our findings regarding the number and general characteristics of available benchmarks (39 benchmarks identified, temporal distribution, and geographic origins) due to the comprehensive database searches across multiple platforms and standardized data extraction procedures. We have moderate certainty in quality assessments and methodological characteristics, as 26% (10/39) of benchmarks received predominantly “cannot tell” ratings on MMAT criteria due to incomplete methodological reporting. We have low certainty in cross-benchmark performance comparisons due to substantial heterogeneity in evaluation metrics, model versions, and reporting standards, which precluded direct quantitative synthesis. These certainty assessments reflect limitations in the primary benchmark literature rather than deficiencies in our review methodology.

### Limitations of Current Clinical Medical LLM Benchmarks

Current clinical medical LLM benchmarks exhibit several systematic limitations that constrain their generalizability and clinical applicability. Geographic bias represents a pervasive challenge, with benchmarks predominantly reflecting Western or East Asian medical practices and health care systems. This geographic concentration limits the applicability of evaluation results to diverse global health care contexts. Furthermore, our quality assessment (Table 2) revealed that 26% (10/39) of included benchmarks provided insufficient methodological detail for proper MMAT evaluation, indicating systemic poor reporting standards in the primary literature that undermine confidence in reported performance metrics.

The dominance of multiple-choice question formats presents another significant limitation. While this format facilitates automated evaluation and standardized scoring, it may not accurately reflect the complexity of real-world clinical decision-making, which often involves open-ended reasoning, differential diagnosis consideration, and nuanced patient communication [17].

Limited continuous update mechanisms further constrain benchmark relevance as medical knowledge rapidly evolves [3,7,69]. Most benchmarks represent static snapshots of medical information rather than dynamic resources that incorporate emerging research findings and updated clinical guidelines. However, some initiatives address these geographical and cultural limitations. TRINDs [37] and AfriMed-QA [36] specifically target underrepresented regions, providing valuable perspectives from diverse health care contexts and addressing gaps in global medical knowledge representation.

### Emerging Themes in Benchmark Evolution

Contemporary medical LLM benchmarks demonstrate increasing sophistication in addressing specialized clinical domains and complex evaluation scenarios. Multimodal capabilities represent a significant advancement, with MedXpertQA incorporating 4460 questions, including 2005 multimodal instances that integrate visual information with textual medical content [64]. This multimodal approach better reflects clinical practice, where physicians routinely interpret medical images alongside patient history and examination findings.

Specialized medical domains receive focused attention through dedicated benchmarks. DentalBench encompasses 36,597 questions across 16 dental subfields [59], providing comprehensive coverage of dental knowledge domains. TCMBench addresses Traditional Chinese Medicine through 5473 carefully curated questions [58], bridging traditional and modern medical approaches. These specialized benchmarks enable targeted evaluation of model performance within specific clinical contexts.

Conversational and interactive evaluation formats emerge as important themes. ChatCoach incorporates 3500 conversations that simulate real clinical interactions [62], while MedDialog provides 3.4 million utterances [42] for evaluating dialogue capabilities in medical contexts. MENTAT contributes 203 mental health care scenarios [66], addressing the critical domain of psychiatric care evaluation.

Bias detection and mitigation receive increased attention through innovative approaches. BiasMedQA demonstrates 10%-26% performance degradation under cognitive bias conditions, quantifying bias impact on clinical reasoning [53]. DiversityMedQA incorporates 2109 questions with demographic perturbations [54], systematically evaluating model sensitivity to patient demographic variations and supporting the development of more equitable health care AI systems.

## Discussion

### Principal Findings

This systematic review achieved its primary objectives by comprehensively characterizing medical LLM benchmarks and revealing significant evaluation gaps. We identified and analyzed 39 benchmarks spanning 2017-2025, categorized them into 3 distinct evaluation paradigms (knowledge-based n=21, 54%, practice-based n=15, 38%, and hybrid n=3, 8%), assessed their methodological quality, and identified important gaps in current assessment approaches. Our most significant finding is the quantification of a substantial knowledge-practice performance gap: while LLMs achieve 84%-90% accuracy on knowledge-based medical examinations, approaching or exceeding physician performance, their practice-based clinical competence drops to 45%-69%, with particularly concerning safety assessment performance at only 40%-50%. This 39-45 percentage point gap between examination scores and clinical capability provides the first systematic evidence that passing medical licensing examinations does not equate to clinical competence for AI systems.

The 3-phase evolution of benchmark development we identified reflects the medical AI field’s maturation from basic knowledge testing toward clinical reality. Our analysis identified 3 distinct evolutionary phases in benchmark development. Early benchmarks from 2017-2020, exemplified by LiveQA [38] and MedicationQA [39], established foundational evaluation methodologies but remained limited to simple factual retrieval. This initial phase parallels early medical education assessment, focusing on memorization rather than application. The intermediate period (2021-2023) witnessed expansion to multimodal assessment and specialized domains, with MedQA [45] introducing 61,097 questions and specialized frameworks such as TCMBench [58] addressing traditional medicine. This expansion mirrors the recognition in medical education that competence requires domain-specific expertise. The current generation (2024-2025) emphasizes ecological validity through frameworks such as MedAgentBench’s FHIR-compliant virtual EHR environment [65] and HealthBench’s 5000 multiturn conversations validated by 262 physicians across 60 countries [16]. This shift toward practice-based evaluation aligns with competency-based medical education reforms, suggesting that AI evaluation is following similar evolutionary patterns as human medical assessment [18].

The safety performance findings carry profound implications for clinical deployment strategies. The CSEDB reveals that even leading models achieve only 40%-50% accuracy on safety-critical scenarios involving contraindication recognition and harm prevention [17]. This performance level falls far below acceptable clinical standards, where medication error rates typically range from 0.1%-1% in well-functioning systems. These results align with FDA guidance emphasizing comprehensive safety evaluation before clinical deployment [8,9]. Our findings directly contradict claims that AI systems are “ready for clinical practice” based solely on examination scores. The inability to reliably identify drug interactions or recognize potentially harmful interventions represents a fundamental barrier to autonomous clinical decision-making, necessitating a paradigm shift from “AI replacement” to “AI augmentation” models, where current deployment strategies should consider maintaining robust human oversight for safety-critical decisions based on current benchmark performance [23].

Geographic representation analysis reveals both progress and persistent inequities in global medical AI development. While our review identified benchmarks spanning 45 languages across 6 continents, representation remains uneven. African and Latin American medical contexts remain significantly underrepresented despite important initiatives such as the AfriMed-QA’s 15,275 questions from pan-African medical schools [36] and TRINDs’ multilanguage evaluation framework [37]. This geographic bias has concrete implications: AI systems trained primarily on Western medical data may fail to recognize diseases endemic to underrepresented regions or misinterpret cultural expressions of illness. This geographic bias limits the global applicability of evaluation results and highlights the need for culturally inclusive assessment methodologies [6,7]. Without addressing these gaps, medical AI risks perpetuating and amplifying existing health care disparities.

The documented “empathy paradox” presents intriguing implications for patient care. Studies consistently demonstrate AI systems outperforming physicians on empathy metrics and patient satisfaction scores [13]. This finding challenges traditional assumptions about human superiority in emotional intelligence and suggests potential complementary roles where AI handles routine empathetic communication while physicians focus on complex emotional support. This apparent advantage in communication and emotional support, coupled with high performance on knowledge retrieval tasks, suggests potential applications in patient education and communication support. However, the paradox also raises concerns: high empathy scores may mask AI’s inability to truly understand patient suffering or make value-based judgments essential in medicine. These strengths must be balanced against the significant gaps in clinical reasoning and safety assessments revealed by practice benchmarks [2,3].

Our quantification of the knowledge-practice gap substantially extends previous systematic reviews and provides actionable metrics for stakeholders. While Shool et al [7] identified heterogeneity in LLM evaluation methodologies, our work reveals specific performance degradation patterns that can guide deployment decisions: practice-based benchmarks (45%-69%) consistently underperform knowledge-based benchmarks (84%-90%). This finding directly refutes the common assumption that high examination scores predict clinical competence—an assumption that has driven premature deployment initiatives. This degradation pattern varies predictably by task complexity (Table 3): factual retrieval maintains 85%-93% accuracy [45], clinical reasoning drops to 50%-60% [56,58], diagnostic tasks achieve 45%-55% success [68], and safety assessment shows the most severe challenges at 40%-50% accuracy [17]. These gradients provide a framework for staged deployment: starting with low-risk factual support tasks before progressing to more complex clinical applications.

### Limitations

Our analysis faces several methodological and scope limitations that readers should consider when interpreting findings. While our review focused specifically on clinical benchmarks, we acknowledge that numerous influential LLM evaluation frameworks exist in nonclinical domains. Notable examples include Holistic Evaluation of Language Models, which evaluates 30+ models across 42 scenarios [70]; BIG-bench with over 200 diverse tasks [71]; MMLU (Massive Multitask Language Understanding) covering 57 subjects [43]; and MT-Bench for conversational evaluation [72]. The exclusion of these frameworks, while necessary for clinical focus, may have overlooked transferable evaluation methodologies. These comprehensive frameworks were excluded as they lacked clinical validation or medical-specific content. However, their methodological rigor in standardized evaluation protocols and systematic assessment of model limitations offers valuable insights for future clinical LLM benchmark development.

The quality assessment revealed concerning gaps in benchmark documentation that limit confidence in reported results. Quality assessment using MMAT criteria revealed variable methodological reporting, with 26% (10/39) of benchmarks providing insufficient detail for proper evaluation—a finding that undermines the validity of performance claims based on these benchmarks. Several benchmarks lacked sufficient detail for reproducibility assessment [31]. This documentation gap means that apparent performance differences between benchmarks may reflect methodological variations rather than true capability differences.

Technical and temporal limitations further constrain our conclusions. The heterogeneity of evaluation metrics complicated direct performance comparisons across benchmarks, potentially masking important performance patterns or creating artificial disparities. The rapid pace of benchmark development means newer frameworks may exist beyond our August 2025 search cutoff. Most importantly, current benchmarks evaluate models in isolation rather than integrated clinical workflows, missing crucial aspects of human-AI collaboration that characterize real-world implementation [18]. This isolation means our performance estimates may underestimate (due to lack of human support) or overestimate (due to controlled conditions) real-world performance. Additionally, static evaluation datasets risk obsolescence as medical knowledge rapidly evolves, with most benchmarks representing fixed snapshots rather than continuously updated resources [3,70]. These static assessments cannot capture AI systems’ ability to incorporate new medical knowledge—a critical requirement for clinical deployment.

Future directions for medical LLM evaluation emerge clearly from our analysis. Continuous benchmark updating mechanisms are essential to maintain relevance as clinical guidelines evolve and new treatments emerge [3]. Standardization of evaluation metrics would facilitate meaningful cross-benchmark comparisons and support regulatory assessment [8]. Multimodal evaluation incorporating imaging, laboratory data, and clinical documentation, as exemplified by MedXpertQA’s 2005 multimodal questions [65], better reflects clinical practice complexity. Longitudinal evaluation tracking model performance across extended care episodes remains unexplored; yet, medical care inherently involves iterative decision-making across multiple encounters [66]. Finally, team-based assessment methodologies that capture human-AI collaboration dynamics represent an important gap in current evaluation methods [24].

The regulatory implications of our findings are immediate and substantial. The documented performance gap between knowledge and practice benchmarks suggests that current certification approaches based on examination performance are insufficient for ensuring clinical safety [8,9]. Regulatory frameworks should mandate practice-based evaluation demonstrating safety and effectiveness in realistic clinical scenarios before deployment approval. The particular challenges in safety assessment, with models achieving only 40%-50% accuracy on safety-critical scenarios [17], suggest the importance of human oversight for high-risk decisions until benchmarks demonstrate consistent safety performance. Professional organizations should establish consensus standards for medical AI evaluation that prioritize ecological validity and comprehensive safety assessment over simplified knowledge testing.

These findings underscore the importance of human-in-the-loop approaches in current medical AI deployment. Given the lower performance observed in practice-based benchmarks compared to knowledge assessments and particularly concerning safety assessment results [17], maintaining physician oversight remains essential for patient safety. Conversely, AI-in-the-loop configurations present promising opportunities for augmenting clinical capabilities, particularly given the documented empathy paradox where AI systems consistently outperform physicians on communication metrics [13]. The optimal implementation strategy likely involves hybrid approaches: leveraging AI’s superior knowledge retrieval and empathetic communication capabilities while preserving human judgment for complex clinical reasoning and safety-critical decisions. However, current benchmarks’ limitation in evaluating human-AI collaboration dynamics highlights the need for future evaluation methods that assess integrated team performance rather than isolated model capabilities [18,66]. Until practice-based benchmarks demonstrate consistent safety and effectiveness in realistic clinical scenarios, staged deployment with robust human oversight must remain the standard approach to medical AI integration [8,23].

### Conclusions

This systematic review challenges the current concept of medical AI evaluation and deployment. Our quantification of a 39-45 percentage point knowledge-practice performance gap provides definitive evidence that examination-based benchmarks are misleading proxies for clinical competence. These findings mandate immediate action: regulators must shift from knowledge-based certification to practice-oriented validation; health care systems must implement robust human oversight protocols, particularly for safety-critical decisions where AI achieves only 40%-50% accuracy; and AI developers must recognize that different clinical applications require distinct evaluation strategies. The methodological gaps we identified—26% (10/39) of benchmarks lacking adequate documentation—establish an urgent need for standardized reporting requirements. Most importantly, our findings definitively establish that autonomous AI deployment is not currently justifiable. The path forward requires reimagining medical AI evaluation from static knowledge testing to dynamic practice assessment, from isolated model evaluation to integrated human-AI team performance, and from 1-time certification to continuous monitoring. Until practice-based benchmarks demonstrate consistent safety and effectiveness, all implementation strategies must maintain human-in-the-loop oversight as an ethical and clinical imperative for patient safety.

## Appendix

Multimedia Appendix 1 PRISMA checklist.

Multimedia Appendix 2 PRISMA-S checklist.

## Abbreviations

AI: artificial intelligence
BERTScore: Bidirectional Encoder Representations From Transformers Score
BLEU: bilingual evaluation understudy
CSEDB: Clinical Safety-Effectiveness Dual-Track Benchmark
EBMQA: Evidence-Based Medicine Question Answering
EHR: electronic health record
FDA: Food and Drug Administration
FHIR: Fast Healthcare Interoperability Resource
GRADE: Grading of Recommendations Assessment, Development, and Evaluation
JAMA: Journal of the American Medical Association
LLM: large language model
MedHELM: Medical Holistic Evaluation of Language Models
MeSH: Medical Subject Headings
MMAT: Mixed Methods Appraisal Tool
MMLU: Massive Multitask Language Understanding
NEJM: New England Journal of Medicine
PRISMA: Preferred Reporting Items for Systematic Reviews and Meta-Analyses
PRISMA-S: an extension to the Preferred Reporting Items for Systematic Reviews and Meta-Analyses Statement for Reporting Literature Searches in Systematic Reviews
QUADAS-2: Quality Assessment of Diagnostic Accuracy Studies, Version 2
ROUGE: Recall-Oriented Understudy for Gisting Evaluation
ROUGE-L: Recall-Oriented Understudy for Gisting Evaluation-Longest Common Subsequence
SCE: Structured Clinical Examination
USMLE: United States Medical Licensing Examination

## Data Availability

All the data are accessible and available upon reasonable request to the corresponding author.
